# Perceptions of Indian Healthcare Practitioners Regarding the 2022 Outbreak of Monkeypox Disease

**DOI:** 10.7759/cureus.35157

**Published:** 2023-02-18

**Authors:** Joseph Betsy, Sherin George, Nebu George, Baiju KV, Anil Sukumaran

**Affiliations:** 1 Periodontics, Saveetha Dental College and Hospitals, Saveetha Institute of Medical and Technical Sciences, Chennai, IND; 2 Dentistry, Pushpagiri Research Centre, Pushpagiri Institute of Medical Sciences and Research Centre, Thiruvalla, IND; 3 Pediatric and Preventive Dentistry, Pushpagiri College of Dental Sciences, Thiruvalla, India, Thiruvalla, IND; 4 Periodontics, Pushpagiri College of Dental Sciences, Thiruvalla, IND; 5 Statistics, Government College for Women, Thiruvananthapuram, IND; 6 Dentistry, Hamad Medical Corporation, Doha, QAT

**Keywords:** knowledge, healthcare practitioners, mpox, monkeypox virus, monkeypox

## Abstract

Introduction

Although only a few cases of monkeypox have been reported in India so far, it is vital for healthcare practitioners to have sufficient knowledge about its epidemiology, clinical presentation, diagnosis, and management. Therefore, this study aimed to understand the perception of Indian healthcare practitioners regarding the 2022 outbreak of monkeypox disease.

Methods

A cross-sectional survey was conducted among 500 medical and dental practitioners from various regions of Kerala, India. The Chi-squared test for proportion was used to determine the significant difference in the knowledge levels of the participants. Binary logistic regression (multivariate) was used to understand the knowledge of healthcare professionals regarding the 2022 outbreak of monkeypox disease.

Results

A total of 424 healthcare professionals completed this survey. Overall, the level of knowledge was good in 64.9% and poor in 35.1% of the participants (p<0.01). Binary logistic regression analysis did not show any statistical significance (p > 0.05) in terms of demographic characteristics. However, in this sample, participants with 6-10 years of work experience were more likely to have improved knowledge scores (odd's ratio OR 1.764). Similarly, participants between the age of 30-40 years were also likely to have improved knowledge scores (OR: 1.065).

Conclusions

Indian healthcare professionals had an overall good level of knowledge regarding the 2022 outbreak of monkeypox. However, a low level of knowledge was found related to the clinical presentation and immunization of monkeypox. This may be due to the low prevalence of monkeypox in this region.

## Introduction

Over the last few decades, an increase in the global population has brought advances in urbanization and has also witnessed increased cases of zoonotic diseases. Monkeypox virus disease is a zoonotic infection caused by the monkeypox virus, a member of the *Orthopoxvirus* group of viruses [1]. Although initial cases of human monkeypox were reported from the African continent, recent reports were also from developed countries in North America, Asia, and Europe [2]. This alarming trend led to the declaration of monkeypox as a global emergency by the World Health Organization (WHO).

The clinical presentation of monkeypox infection was similar to but less severe than smallpox. The cases reported in 2022 often presented with fever, rash, sweats, chills, lymphadenopathy, headache, stiff neck, red eyes, runny nose, sore throat, cough, wheezing, nausea and/or vomiting, abdominal pain, a scrotal lump, itchy maculopapular rashes, and confusion [3-5]. These cases were presented with [6,7] and without oral manifestations [8]. Generalized lymphadenopathy in monkeypox has been reported as a primary differentiating factor between monkeypox and smallpox.

Monkeypox has been reported in many cases during travel to Africa or contact with a monkeypox virus-infected person [9]. However, many new cases in the current outbreak of 2022 do not give a travel history to Africa or exposure to an infected person [3]. Discontinuation of the smallpox vaccination policy about 30-40 years ago could have caused an overall decline in the immunity of the population to smallpox and similar *Orthopoxvirus* diseases [10]. This could be one significant reason for the resurgence of infections by the monkeypox virus in young adults and small children in 2022 who were unvaccinated against smallpox [11]. Some report increased hospitalization in children below 18 years of age [12].

The surveillance system of many countries took preventive steps to control the transmission of the monkeypox virus [5]. This included isolating and quarantining infected people, preparing beds in several hospitals, and installation of thermal scanning systems at various places. Such a robust system is necessary to enable frontline healthcare workers to screen these cases [5]. India receives visitors from different parts of the world annually, increasing the vulnerability to human monkeypox transmission. Although only a few cases of monkeypox have been reported in India so far, it is crucial for healthcare practitioners to have sufficient knowledge about its epidemiology, clinical presentation, diagnosis, and management. This will help to identify and manage cases of the monkeypox virus efficiently and prevent further disease transmission [13]. Hence, we aimed to understand the perception of Indian healthcare practitioners regarding the 2022 outbreak of monkeypox in terms of their knowledge score.

## Materials and methods

This cross-sectional survey was conducted among medical and dental practitioners from various regions of Kerala, India, during September 1-30, 2022, using a convivence sampling method. We sent invitations to 500 participants to take part in the survey. Members of the Indian Medical Association (IMA) and the Indian Dental Association (IDA) who were interested in participating and were practicing in Kerala, India, were included in the survey. The ethical approval was obtained from the Institutional Ethics Committee, Pushpagiri group of Institutions, Thiruvalla, Kerala, India, College, India (IRB/03/08/2022). 

Survey

The questionnaire was sent to the participants through email and Whatsapp (WhatsApp LLC, Menlo Park, California, United States). The subject heading of the message described the purpose of the survey, while the main content contained the weblink to an anonymous web-based survey. Responses within a one-month time horizon were accepted for the study. Participants' response to the virtual survey was considered implicit consent. The participants were told about the time to complete the survey and data privacy.

Questionnaire

Before disseminating the survey, a questionnaire was drafted based on previous studies and frequently asked questions (FAQ) by WHO. This was pilot tested among 10 medical and dental practitioners, professors, administrators, and experts to determine its validity, following which necessary modifications were made. Cronbach's alpha equal to or more than 0.80 was considered in terms of internal consistency. The survey was finalized, taking into consideration their suggestions and comments. The study is reported based on the strengthening of the reporting of observational studies in epidemiology (STROBE) checklist.

The questionnaire consisted of two parts. The first part collected the sociodemographic details of the respondents. The second part addressed the professional knowledge of the respondents. The second part had 20 questions that could be answered as "yes", or "no".

The following sociodemographic variables were assessed: age (<20/ 20-30/ 31-40/ >40 years), gender (male/female), years of work experience (0-5/ 6-10/ 11-15/ >15 years), specialty (dental/medical), employment sector (teaching institutes/ clinical practice), and the number of years after graduation/postgraduation (0-5/ 6-10/ 11-15/ >15 years).

The second section gathered the knowledge of these practitioners regarding the 2022 outbreak of monkeypox through 20 questions.

Sample size

Since no previous study evaluated the perception of healthcare practitioners about monkeypox, a power analysis was done based on the previous study by Harapan et al. in 2020 [3]. Assuming that 50% of the medical and dental practitioners would have a good knowledge of monkeypox, 382 respondents were required as the minimum sample size (5% margin of error and confidence interval of 95%).

Statistical analysis

The data was imported and analyzed using IBM SPSS Statistics for Windows, Version 20.0 (Released 2011; IBM Corp., Armonk, New York, United States). Frequencies and percentages were used as summary statistics. The chi-squared test for proportion was used to determine the significant difference in the awareness levels of the participants. Binary logistic regression (multivariate) was used to understand the knowledge of health professionals regarding the 2022 outbreak of monkeypox.

## Results

Participants (demographics)

A total of 424 healthcare professionals completed this survey. The demographic details of the participants are shown in Table 1. It can be seen that the majority (70.3%) of the participants were between the ages of 20 and 30, followed by 13.9% who were older than 40 years. The least number of participants was below 20 years (7.8%).

**Table 1:** Sociodemographic characteristics of the participants (N=424)

Variables	Categories	Frequency	Percent
Age (years)			
	<20	33	7.8
	20-30	298	70.3
	31-40	34	8.0
	>40	59	13.9
Sex			
	Female	297	70.0
	Male	127	30.0
Years of work experience (years)			
	<5	329	77.6
	6-10	25	5.9
	11-15	18	4.2
	>15	52	12.3
Speciality			
	Dental	192	45.3
	Medical	232	54.7
Employment sector			
	Teaching institute	121	28.5
	Practice	303	71.5
Number of years after graduation/postgraduation (years)			
	<5	342	80.7
	6-10	18	4.2
	11-15	18	4.2
	>15	46	10.9
Total		424	100.0

Knowledge of health professionals regarding the 2022 outbreak of monkeypox

Table 2 shows that out of the 20 questions, three were answered correctly by over 70% of participants. These questions were: (i) "All healthcare professionals should implement standard infection control precautions with suspected cases of monkeypox" (85.8% answered correctly), (ii) "Monkeypox has a very high fatality rate of above 50%" (73.8% answered correctly), and (iii) "N95 masks and other personal protective equipment (PPE) are of no value when coming into close contact with a case of monkeypox" (70.3% answered correctly).

**Table 2:** Knowledge of health professionals regarding the 2022 outbreak of monkeypox disease (N=424) PPE: personal protective equipment; NS: Not Significant (P>0.05) * Significant at 5% (P<0.05) **: Significant at 1% (P<0.01)

Questions	Response Category	Frequency	Percent	Chi-square value	P value
The vesicular lesions in monkeypox are broad and non-itchy	Incorrect	295	69.6	64.991	0.000**
	Correct	129	30.4		
Monkeypox has a very high fatality rate of above 50%	Incorrect	111	26.2	96.236	0.000**
	Correct	313	73.8		
All healthcare professionals should implement standard infection control precautions with suspected cases of monkeypox	Incorrect	60	14.2	217.962	0.000**
	Correct	364	85.8		
Oral mucosa and lips are not at all affected in monkeypox	Incorrect	164	38.7	21.736	0.000**
	Correct	260	61.3		
The traditional smallpox vaccine is protective against monkeypox	Incorrect	302	71.2	76.415	0.000**
	Correct	122	28.8		
Lesions do not appear in the hand and eyes in monkeypox	Incorrect	174	41.0	13.623	0.000**
	Correct	250	59.0		
Lesions may originate in the genital region as per the observations of current outbreaks of monkeypox	Incorrect	215	50.7	0.085	0.771NS
	Correct	209	49.3		
The lesions in monkeypox are smaller than those in chickenpox	Incorrect	179	42.2	10.274	0.001**
	Correct	245	57.8		
There is more chance of contracting monkeypox if an individual has already been vaccinated with the smallpox vaccine	Incorrect	218	51.4	0.340	0.560NS
	Correct	206	48.6		
The reverse transcription-polymerase chain reaction test is recommended for identifying the monkeypox virus during an acute infection	Incorrect	210	49.5	0.038	0.846NS
	Correct	214	50.5		
Immunosuppressed people, pregnant women, and children younger than 12 years do not have an increased risk for monkeypox	Incorrect	137	32.3	53.066	0.000**
	Correct	287	67.7		
Management of monkeypox is essentially symptomatic	Incorrect	171	40.3	15.858	0.000**
	Correct	253	59.7		
Specific antiviral agents are available that completely cure monkeypox	Incorrect	199	46.9	15.858	0.000**
	Correct	225	53.1		
Smallpox vaccine against monkeypox could be prescribed for patients with comorbidities	Incorrect	303	71.5	78.123	0.000**
	Correct	121	28.5		
Passive immunization with immune globulin vaccinia is successful in the case of monkeypox	Incorrect	283	66.7	47.557	0.000**
	Correct	141	33.3		
Vaccines against smallpox are effective in preventing monkeypox and postexposure prophylaxis	Incorrect	265	62.5	26.500	0.000**
	Correct	159	37.5		
N95 masks and other PPE are of no value when coming into close contact with a case of monkeypox	Incorrect	126	29.7	69.774	0.000**
	Correct	298	70.3		
Monkeypox spreads through close contact and respiratory droplets	Incorrect	85	20.0	152.160	0.000**
	Correct	339	80.0		
Commercial tests to detect monkeypox are available at this point in India.	Incorrect	149	35.1	37.443	0.000**
	Correct	275	64.9		
Homosexuals do not have a higher risk of monkeypox	Incorrect	188	44.3	5.434	0.020*
	Correct	236	55.7		

Seven questions were answered correctly by less than 50% of the participants. These questions were: (i) The vesicular lesions in monkeypox are broad and non-itchy (30.4% answered correctly), (ii) The traditional smallpox vaccine is protective against monkeypox (28.8% answered correctly), (iii) Lesions may originate in the genital region as per the observations of current outbreaks of monkeypox (49.3% answered correctly), (iv) There is more chance of contracting monkeypox if an individual has already been vaccinated with the smallpox vaccine (48.6% answered correctly), (v) Smallpox vaccine against monkeypox could be prescribed for patients with comorbidities (28.5% answered correctly), (vi) Passive immunization with immune globulin vaccinia is successful in the case of monkeypox (33.3% answered correctly), nd (vii) Vaccines against smallpox are effective in preventing monkeypox and postexposure prophylaxis (37.5% answered correctly).

There was a statistically significant difference between the responses of these participants (p<0.01) except for four questions (p>0.05). The questions for which the participants lacked clarity (p>0.05) were: (i) Lesions may originate in the genital region as per the observations of current outbreaks of monkeypox (p=0.771), (ii) There is more chance of contracting monkeypox if an individual has already been vaccinated with the smallpox vaccine (p=0.560), (iii) The reverse transcription-polymerase chain reaction test is recommended for identifying the monkeypox virus during acute infection (p=0.846), and (iv) Specific antiviral agents are available that completely cure monkeypox (p=0.207). Overall, the level of awareness was good in 64.9% and poor in 35.1% of the participants (p<0.01).

Regression analysis for knowledge regarding the 2022 outbreak of monkeypox

Table 3 shows a binary logistic regression analysis's outcome when considering participants' knowledge regarding the 2022 outbreak of monkeypox in either a "Yes" or "No" answer. Statistical significance was not found (p > 0.05) in this logistic regression model in terms of demographic characteristics such as age, sex, years of work experience, specialty (medical or dental), employment sector (teaching institute or clinical practice), and the number of years after graduation/ postgraduation.

**Table 3:** Binary logistic regression for knowledge of health professionals regarding the 2022 outbreak of monkeypox disease (N=424) NS: Not Significant (P>0.05)

Variables	Knowledge		B	Odds ratio	95% CI	P-value
	Incorrect	Correct				
Age (years)						
<20	24(72.7)	9(27.3)	-1.277	0.279	0.046-1.692	0.165^NS^
20-30	205(68.8)	93(31.2)	-1.150	0.317	0.062-1.617	0.167^ NS^
30-40	17(50.0)	17(50.0)	0.063	1.065	0.274-4.129	0.928^ NS^
>40	27(45.8)	32(54.2)	-	Reference	-	-
Sex						
Female	201(67.7)	96(32.3)	-0.262	0.770	0.482-1.229	0.273^ NS^
Male	72(56.7)	55(43.3)	-	Reference	-	-
Years of work experience (years)						
<5	227(69.0)	102(31.0)	-0.128	0.880	0.168-4.605	0.879^ NS^
6-10	12(48.0)	13(52.0)	0.568	1.764	0.330-9.427	0.507^ NS^
11-15	10(55.6)	8(44.4)	-0.201	0.818	0.136-4.909	0.826^ NS^
>15	24(46.2)	28(53.8)	-	Reference	-	-
Specialty						
Dental	125(65.1)	67(34.9)	-0.363	0.695	0.437-1.107	0.126^ NS^
Medical	148(63.8)	84(36.2)	-	Reference	-	-
Employment sector						
Teaching Institute	79(65.3)	42(34.7)	-0.304	0.738	0.455-1.196	0.217^ NS^
Practice	194(64.0)	109(36.0)	-	Reference	-	-
Number of years after graduation/Post graduation (years)						
<5	233(68.1)	109(31.9)	-0.005	0.995	0.158-6.279	0.996^ NS^
6-10	11(61.1)	7(38.9)	-1.146	0.318	0.056-1.810	0.196^ NS^
11-15	9(50.0)	9(50.0)	-0.395	0.674	0.116-3.930	0.661^ NS^
>15	20(43.5)	26(56.5)	-	Reference	-	-

However, in this sample, participants with 6-10 years of work experience were more likely to have improved knowledge scores (OR 1.764). Similarly, participants aged 30-40 years were also likely to have improved knowledge scores (OR: 1.065).

## Discussion

Knowledge of health professionals regarding the 2022 outbreak of monkeypox

Monkeypox disease has spread in multiple regions and was declared a "global emergency" by WHO. The regions of southeast Asia have been on high alert for monkeypox since the first case was reported in India in July 2022 [14]. It was reported by a 35-year-old man with a history of travel from the Middle East earlier that week. Since then, all countries have taken preventive measures to control the spread of monkeypox.

The perceptions of healthcare practitioners impact the healthcare sector's outlook towards it [15]. In the present study, an overall good level of awareness was seen in the majority (64.9%) of the participants. A recent study among dental professionals in India found a low level of knowledge about monkeypox [16]. They reported that 24.8% of the participants were unaware of monkeypox. In our study, 61.3% had knowledge about oral lesions of monkeypox, and 59.0% had knowledge about other clinical presentations. Contrary to our findings, only 31.2% of subjects had knowledge regarding oral manifestations of the disease in another recent study [16]. Another study also reports low levels of knowledge scores among general practitioners in Indonesia [13].

The good level of knowledge in the present study could be because the majority (70.3%) of the participants were between the ages of 20 and 30 who usually have good access to the internet and frequently used social media. A recent study showed higher education levels and working profiles increased knowledge levels [16]. In these participants, online media (internet) was the most preferred (42.2%) source of information [16]. Another study done on the general population also found that three-fourths of the participants used social media as their main source of information [17]. The availability of scientific data in online academic journals may have a better influence on healthcare professionals’ knowledge regarding the disease.

It was interesting to note that most of the participants (85.8%) of the present study were aware of the need to use standard infection control procedures, including the use of N95 masks and other PPE, with a suspected case of monkeypox (70.3%). Similarly, most participants (73.8%) also knew that monkeypox does not have a high fatality rate.

A low level of knowledge was found among the present study participants related to the clinical presentations of monkeypox, the origin of lesions, the effectiveness of immunization with smallpox vaccines against monkeypox, diagnosis of monkeypox, and available treatment for monkeypox. The low prevalence of monkeypox cases in this region could be a probable reason. A recent study reported 44.8% of participants had the wrong information about the resemblance of monkeypox with smallpox disease [16]. Furthermore, only 26.2% of the respondents in a Jordanian health school survey had knowledge about the availability of vaccination to prevent monkeypox [18].

An earlier online survey conducted on general practitioners in Indonesia showed 36.5% of them had good knowledge when a 70% cutoff point for the knowledge domain was applied [13]. Within this cut-off, lower knowledge was found in those older than 30 years (adjusted OR (aOR): 0.61; 95%CI: 0.39-0.96, p = 0.033). Our results are contrary to this study as participants between the age of 30 to 40 years were likely to have improved knowledge scores (OR: 1.065; 95%CI: 0.274-4.129, p=0.928).

Another interesting finding was that working in teaching institutes or clinical practice did not influence the knowledge levels of the participants of the present study. But an earlier study reports general practitioners working in private clinics had less knowledge compared to general practitioners in community health centers (aOR: 0.55; 95%CI: 0.31--0.99, p = 0.047) [13].

In our study, logistic regression showed that the knowledge of medical and dental practitioners regarding the 2022 outbreak of monkeypox disease was not influenced by demographic characteristics such as age, sex, years of work experience, specialty (medical or dental), employment sector (teaching institute or clinical practice), and the number of years after graduation/ postgraduation. However, in this sample, work experience of 6-10 years and age between 30 to 40 years seem to influence knowledge scores. Similarly, in a study done on students in Jordanian health schools, age was significantly associated with improved monkeypox knowledge for most of the participants [18].

Strengths and limitations of the study

This is the first study that reports the perception of medical and dental professionals regarding the outbreak of the 2022 monkeypox disease in India. However, this is a cross-sectional study. Therefore, changes in their perception over time cannot be provided. This is one of the limitations of this study. Moreover, we did not categorize medical and dental professionals based on their specialty and region of work. Since the participants were from different backgrounds, the findings of this study may lack generalizability.

Recommendations

There are certain precautions to be taken in dental and medical clinics. Transmission of the monkeypox virus can be prevented in clinical settings by taking standard infection control precautions when treating patients with monkeypox symptoms [19-22]. Proper contact and droplet infection control should also be practiced. The patient should be isolated during examination and treatment. Exposed skin lesions should be covered, and the patient's nose and mouth be covered using a surgical mask. Elective dental treatment should be postponed in patients with probable or confirmed monkeypox infection until the patient is non-infectious [23]. Oral lesions [24] and ocular lesions [25] are possible complications of monkeypox. If not careful, ocular monkeypox can potentially cause conjunctivitis and even vision loss. Good hand hygiene should be practiced to reduce the risk of auto-inoculation.

The knowledge of healthcare professionals can further be improved through educational programs and training in identifying and managing monkeypox. Health professionals should be given information regarding the clinical presentations of monkeypox, the origin of lesions, the effectiveness of immunization with smallpox vaccines against monkeypox, diagnosis of monkeypox, and available treatment for monkeypox. Based on the findings of this study, increasing the knowledge about these factors can improve the overall knowledge score of healthcare practitioners regarding monkeypox.

## Conclusions

Within the study's limitations, it can be concluded that the Indian healthcare practitioners had an overall good level of awareness regarding the 2022 outbreak of monkeypox in terms of their knowledge score. There was good knowledge about oral lesions of monkeypox. However, a low level of knowledge was found related to the clinical presentation and immunization of monkeypox. This may be due to the low prevalence of monkeypox in this region. Educational programs and training in identifying and managing monkeypox disease are recommended for healthcare professionals.

## Appendices

**Table 4:** Questionnaire to assess the knowledge of health professionals regarding the 2022 outbreak of monkeypox disease mpox: monkeypox

Perceptions of Indian healthcare practitioners regarding the 2022 outbreak of monkeypox disease		
Sociodemographic details		Response
Age (in years)		
Sex	Female Male	
Years of work experience	<5 years 6-10 years 11-15 years >15 years	
Specialty	Dental Medical	
Employment sector	Teaching institute Practice	
Number of years after graduation/postgraduation	<5 years 6-10 years 11-15 years >15 years	
There are 20 questions about monkeypox (mpox). Please read each question carefully and mark the options as: 1=YES, 2=NO		
Questions		Options (1=YES, 2=NO)
The vesicular lesions in mpox are broad and non-itchy.		
Mpox has a very high fatality rate of above 50%.		
All healthcare professionals should implement standard infection control precautions with suspected cases of mpox.		
Oral mucosa and lips are not at all affected in mpox.		
The traditional smallpox vaccine is protective against mpox.		
Lesions do not appear in the hand and eyes in mpox.		
Lesions may originate in the genital region as per the observations of current outbreaks of mpox.		
The lesions in mpox are smaller than those in chickenpox.		
There is more chance of contracting mpox if an individual has already been vaccinated with the smallpox vaccine.		
The reverse transcription-polymerase chain reaction test is recommended for identifying the mpox virus during an acute infection.		
Immunosuppressed people, pregnant women, and children younger than 12 years do not have an increased risk for mpox.		
Management of mpox is essentially symptomatic.		
Specific antiviral agents are available that completely cure mpox.		
Smallpox vaccine against mpox could be prescribed for patients with comorbidities.		
Passive immunization with immune globulin vaccinia is successful in the case of mpox		
Vaccines against smallpox are effective in preventing mpox and postexposure prophylaxis.		
N95 masks and other personal protective equipment (PPE) are of no value when coming into close contact with a case of mpox		
Mpox spreads through close contact and respiratory droplets.		
Commercial tests to detect mpox are available at this point in India.		
Homosexuals do not have a higher risk of mpox.		
